# High level of pre-treatment C-reactive protein to albumin ratio predicts inferior prognosis in diffuse large B-cell lymphoma

**DOI:** 10.1038/s41598-021-82087-6

**Published:** 2021-01-29

**Authors:** Jongheon Jung, Hyewon Lee, Ja Yoon Heo, Myung Hee Chang, Eunyoung Lee, Weon Seo Park, Ju-Hyun Park, Hyeon-Seok Eom

**Affiliations:** 1 Center for Hematologic Malignancy, National Cancer Center, 323 Ilsan-ro, Ilsandong-gu, Goyang, Geyonggi 410-769 Republic of Korea; 2 Yonsei University College of Medicine, Seoul, Republic of Korea; 3 Division of Oncology-HematologyDepartment of Internal Medicine, National Health Insurance Service Ilsan Hospital, Ilsan, Republic of Korea; 4 Department of Pathology, National Cancer Center, Goyang, Republic of Korea; 5 Department of Statistics, Dongguk University, Seoul, Republic of Korea

**Keywords:** Cancer, Biomarkers, Oncology

## Abstract

The C-reactive protein-to-albumin ratio (CAR) has not been assessed in diffuse large B cell lymphoma (DLBCL, the most common non-Hodgkin lymphoma). This retrospective study evaluated the prognostic value of CAR in 186 DLBCL patients. A CAR value of 0.158 was selected as the most discriminative cut-off for identifying patients with high CAR values (73/141 patients, 51.8%). During a median follow-up of 32.5 months, the high CAR group had significantly poorer complete response to induction therapy (64.4% vs. 92.6%; p < 0.001), 3-year overall survival (OS) (68.3% vs. 96.2%; p < 0.0001), and 3-year progression-free survival (PFS) (53.5% vs. 88.0%; p < 0.0001). After adjusting for the International Prognostic Index components, a high CAR value independently predicted poor OS (HR: 6.02, 95% CI 1.19–30.38; p = 0.030) and PFS (HR: 3.62, 95% CI 1.40–9.36; p = 0.008). In an independent validation cohort (n = 50), patients with CAR > 0.158 also showed worse 3-year OS (47.9% vs. 87.2%, p = 0.0035) and 3-year PFS (36.1% vs. 82.1%, p = 0.0011). A high CAR remained significantly associated with poor outcomes for > 60-year-old patients (OS: p = 0.0038, PFS: p = 0.0015) and younger patients (OS: p = 0.0041, PFS: p = 0.0044). Among older patients, a high CAR value also predicted non-relapse mortality (p = 0.035). Therefore, the CAR might complement the International Prognostic Index in DLBCL cases.

## Introduction

Diffuse large B cell lymphoma (DLBCL) is the most common highly aggressive non-Hodgkin lymphoma^1,2^, and the initial treatment is often standard induction chemotherapy using rituximab, cyclophosphamide, doxorubicin, vincristine, and prednisone (R-CHOP)^3^. In addition, high-dose chemotherapy plus autologous hematopoietic stem cell transplantation (HSCT) has been frequently used for relatively young patients with relapsed or refractory DLBCL^4^. However, a large proportion of patients still die because of relapsed DLBCL^5,6^. Therefore, it is still important to predict the prognosis of patients with DLBCL.

The International Prognostic Index (IPI) is a useful prognostic marker for DLBCL, and some variants have been used to incorporate rituximab into standard chemotherapy as well as to consider more specific clinical factors, such as age, serum lactate dehydrogenase (LDH), and cancer stage^7–9^. Immunohistochemical markers, such as CD10, BCL6, and MUM1, have been used to clinically differentiate between the germinal centre B cell type (GCB) and non-GCB type, which revealed that the GCB type was associated with superior outcomes^10^. However, there is still a need for simple markers that can predict the prognosis of patients who experience relapse after remission or who fail to respond to immunochemotherapy.

Various markers related to the tumour microenvironment may be used in prognostication, including neutrophils, lymphocytes, platelets, serum globulin, ferritin, and serum free light chain (FLC)^11–14^. A common inflammatory marker is C-reactive protein (CRP), which may be clinically relevant in various malignancies^15,16^. Previous studies have also revealed that serum albumin can reflect the patient’s nutritional status and that hypoalbuminemia might indicate the presence of cancer-related inflammation^17,18^. Thus, the C-reactive protein-to-albumin ratio (CAR) has been suggested as an easily accessible parameter that might facilitate prognostication for various malignancies, including lung cancer, gastric cancer, and colorectal cancer^19–21^. However, the CAR has not been evaluated for haematological malignancies. Thus, the present study aimed to examine the prognostic value of the CAR and other laboratory biomarkers in DLBCL.

## Patients and methods

### Patients

This retrospective study evaluated 186 patients with histologically diagnosed DLBCL who received R-CHOP between 2006 and 2018 at the National Cancer Center in Korea. The inclusion criteria were age of ≥ 20 years at the diagnosis, received at least one cycle of R-CHOP as induction therapy, and available baseline clinical and laboratory data from before the initial therapy. The exclusion criteria were age of < 20 years at the diagnosis, primary central nervous system DLBCL, received other induction therapy (including radiation treatment or no chemotherapy), and immunodeficiency (including human immunodeficiency virus seropositivity). The medical records of the 186 patients were reviewed, and we ultimately identified 141 patients with baseline data for determining their CAR. Molecular subtypes were determined based on immunohistochemistry results according to the criteria of Hans et al.^10^. The initial responses to induction therapy were evaluated based on the Lugano classification, which classifies responses as complete response (CR), partial response (PR), stable disease (SD), and progressive disease (PD)^22^. The independent validation cohort included 50 patients who were treated with the identical regimen between 2015 and 2018 at the National Health Insurance Service Ilsan Hospital, Korea. The same inclusion/exclusion criteria and response evaluation were applied for that study.

### Laboratory testing

The pre-treatment CAR was determined using laboratory results from the day closest to the start of the R-CHOP treatment (< 4 weeks). The CAR values were calculated as the serum CRP level (reference range: 0–0.30 mg/dL) divided by the serum albumin level (reference range: 3.3–5.2 g/dL). Serum globulin was measured based on the difference between the serum levels of protein and albumin (reference range: 2.2–3.4 g/dL), and the albumin-to-globulin ratio (AGR) was calculated as serum albumin divided by serum globulin. We also obtained data regarding serum ferritin levels (reference range: 16–400 ng/mL) and beta 2 microglobulin levels (reference range: 0.61–2.37 mg/L). All laboratory data had been obtained using the XE-2100 system (Sysmex, Kobe, Japan) at the National Cancer Center, Korea and at the National Health Insurance Service Ilsan Hospital, Korea.

### Statistical analysis

Overall survival (OS) was calculated from the first day of induction therapy to death from any cause or the date of the last follow-up. Progression-free survival (PFS) was calculated from the start of induction therapy to the earliest instance of disease progression or death. Non-relapse mortality (NRM) was defined as the time from the start of induction therapy to death without relapse. To identify an appropriate cut-off value for CAR in patients with DLBCL, we used one of Budczies et al.’s cut-off finder methods^23^; this identifies the optimal cut-off for the CAR value, at which the split results in the most significant log-rank test from survival analysis. To avoid any split with an extremely low or high CAR value, the method was employed under the constraint that either low or high CAR groups are assigned at least 20% of the total sample size. As a result, it was revealed that a CAR value of 0.158 was the most discriminative cut-off. The same method was used to identify appropriate cut-off values for the AGR, serum ferritin, and beta 2 microglobulin.

The high and low CAR groups were compared to identify differences in clinical characteristics and outcomes, including the response rate, OS, and PFS. Continuous variables were analysed using the two-sample t-test or the Mann–Whitney U test, depending on the result of the normality test. Categorical variables were analysed using the χ^2^ test or Fisher’s exact test, as appropriate. The survival analyses were performed using the Kaplan–Meier method and the log-rank test. Univariable and multivariable Cox proportional hazard models were used to evaluate each variable’s prognostic value, and the results were reported as hazard ratios (HRs) and 95% confidence intervals (CIs). All statistical analyses were performed using R software (version 3.5.1; R Foundation for Statistical Computing, Vienna, Austria), and differences were considered significant at two-sided *p*-values of < 0.05.

### Ethical approval

The retrospective protocol was approved by the institutional review board (IRB) (National Cancer Center 2020-0110, National Health Insurance Service Ilsan Hospital 2020-04-020) and complied with the Declaration of Helsinki. Written informed consent was exempted from IRB because no intervention was involved due to the nature of the retrospective study.

### Conference presentation

The abstract was presented in the session for oral and poster abstracts at the 60th annual American Society of Hematology meeting in 2018.

## Results

### Patient characteristics

The 141 patients with data available to calculate their CAR (pre-treatment CRP and albumin values) were assigned to the high CAR group (73 patients, 51.8%) or the low CAR group (68 patients, 48.2%). All patients had histologically confirmed DLBCL and received at least one cycle of induction R-CHOP therapy. The patients’ baseline characteristics are shown in Table 1. The high and low CAR groups had similar proportions of male sex (42 patients [57.5%] vs. 37 patients [54.4%]), and most patients in both groups were > 60 years old at the diagnosis, with no significant difference in the age distributions (p = 0.867). The high CAR group had a significantly higher proportion of patients with high IPI scores of 4–5 (21 patients [28.8%] vs. 6 patients [8.8%]), and most of the IPI components were poorer in the high CAR group, with the exception of age of > 60 years. The characteristics of validation cohort are shown in Supplementary Table S1, in which 30 (60.0%) patients were classified as the high CAR group.

**Table 1:** Clinical characteristics of patients with diffuse large B-cell lymphoma.

	Total	Low CAR (≤ 0.158)	High CAR (> 0.158)	P*
Number	141	68 (48.2%)	73 (51.8%)	
Male sex	79 (56.0%)	37 (54.4%)	42 (57.5%)	0.737
Age > 60 years	74 (52.5%)	35 (51.5%)	39 (53.4%)	0.867
Stage > 2	79 (56.0%)	23 (33.8%)	56 (76.7%)	** < 0.001**
ECOG PS > 1	19 (13.5%)	3 (4.4%)	16 (21.9%)	***0.003***
LDH > UNL	90 (63.8%)	28 (41.2%)	62 (84.9%)	** < 0.001**
Extranodal > 1	49 (34.8%)	13 (19.1%)	36 (49.3%)	** < 0.001**
**IPI**				** < 0.001**
0	17 (12.1%)	13 (19.1%)	4 (5.5%)	
1	32 (22.7%)	26 (38.2%)	6 (8.2%)	
2	29 (20.6%)	17 (25.0%)	12 (16.4%)	
3	36 (25.5%)	6 (8.8%)	30 (41.1%)	
4	22 (15.6%)	6 (8.8%)	16 (21.9%)	
5	5 (3.5%)	0	5 (6.8%)	
Non-GCB type	102 (79.1%)	46 (73.0%)	56 (84.8%)	0.130
Cycles of R-CHOP	6^1–8^	6^1–8^	6^1–8^	0.395
**Response to induction**				
CR	110 (78.0%)	63 (92.6%)	47 (64.4%)	** < 0.001**^†^
PR	10 (7.1%)	2 (2.9%)	8 (11.0%)	
SD	1 (0.7%)	0	1 (1.4%)	
PD	8 (5.7%)	0	8 (11.0%)	
Not available	12 (8.5%)	3 (4.4%)	9 (12.3%)	
Overall response	120 (93.0%)	65 (100%)	55 (85.9%)	***0.001***
CRP (mg/dL)	0.65 [0.01–18.03]	0.2 [0.01–0.64]	2.2 [0.54–18.03]	** < 0.001**
Albumin (g/dL)	3.9 [2.3–4.9]	4.1 [3.3–4.9]	3.6 [2.3–4.6]	** < 0.001**
Globulin (g/dL)	2.6 [1.5–4.3]	2.6 [1.7–3.8]	2.6 [1.5–4.3]	0.799
Ferritin (ng/mL)	98 [3–11,700]	74 [3–963]	149 [10–11,700]	***0.018***
B2-microglobulin (mg/L)	2.2 [1.2–11.1]	2.1 [1.2–4.6]	2.7 [1.5–11.1]	** < 0.001**

Data are presented as number of patients (frequency) for categorical variables and as median [range] for continuous variables.

*CAR* C-reactive protein-to-albumin ratio, *ECOG PS* Eastern Cooperative Oncology Group performance status, *LDH* lactate dehydrogenase, *UNL* upper normal limit, *IPI* International Prognostic Index, *GCB* germinal centre B-cell, *R-CHOP* rituximab, cyclophosphamide, doxorubicin, vincristine, and prednisone, *CR* complete response, *PR* partial response, *SD* stable disease, *PD* progressive disease.

*High-CAR group vs. low-CAR group.

^†^CR vs. others.

Although 12 patients were excluded because of missing data regarding GCB status, the results revealed that the non-GCB type accounted for 56 cases (84.8%) in the high CAR group and 46 cases (73.0%) in the low CAR group. The high and low CAR groups both had a median of 6 cycles of induction chemotherapy (p = 0.395), although the high CAR group had significantly poorer CR to induction R-CHOP therapy (64.4% vs. 92.6%; p < 0.001). When we considered the overall response (CR + PR), the low CAR group had significantly better response to induction therapy (100% vs. 85.9%; p = 0.001). During the follow-up period, we identified 5 deaths in the low CAR group and 20 deaths in the high CAR group. Fifteen deaths were related to infectious diseases, such as pneumonia, neutropenic fever, and septic shock. Five deaths were related to disease progression, and 5 deaths were related to other causes, such as brain haemorrhage, pneumoperitoneum, and liver failure. Among the 116 surviving patients, 95 patients have been censored during follow-up at the time of analysis and 21 patients have been censored based on loss to follow-up.

### Clinical outcomes according to the CAR

**Figure 1:**
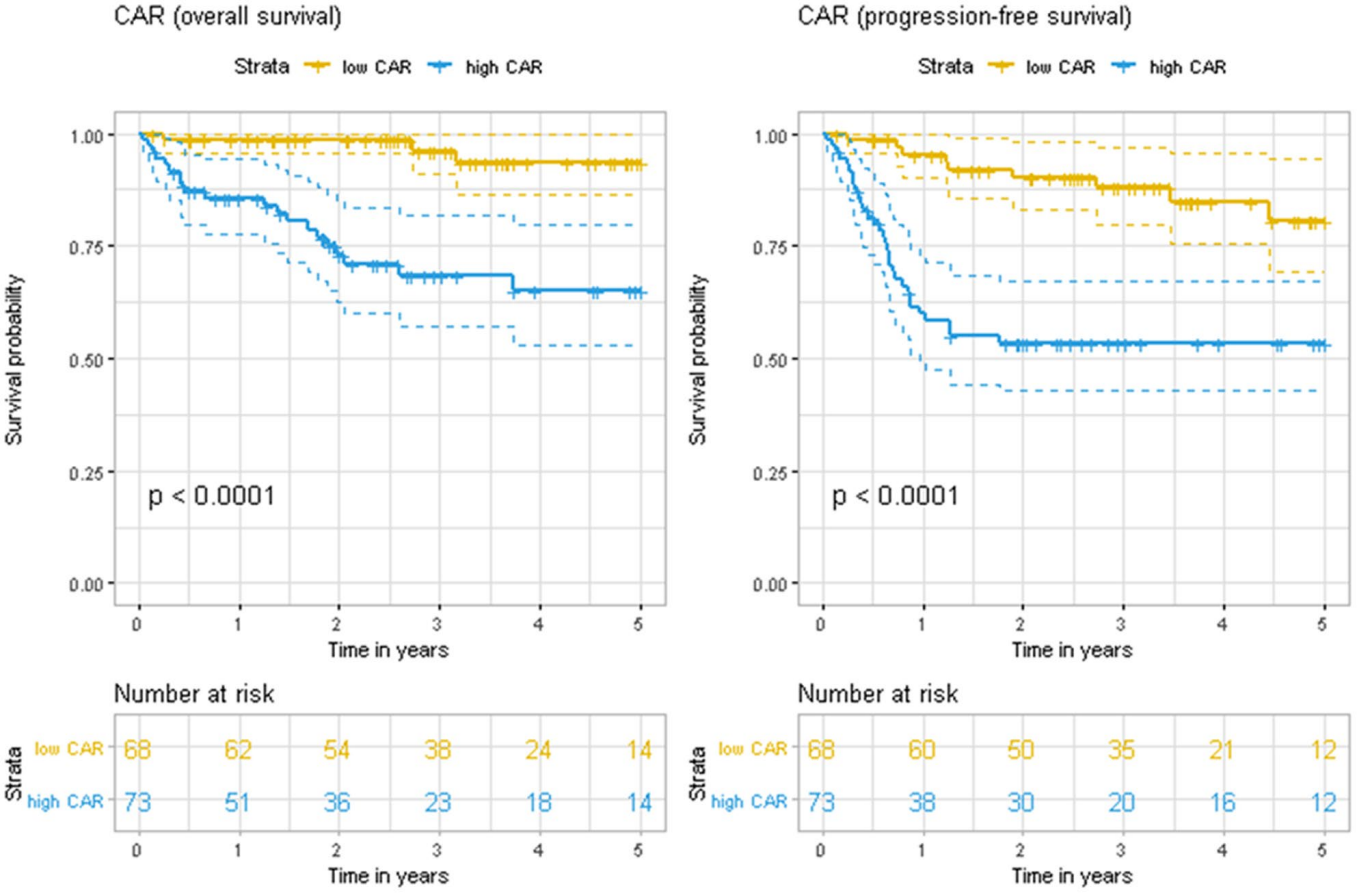
Kaplan–Meier curves for overall survival and progression-free survival according to the C-reactive protein-to-albumin ratio (CAR) at the diagnosis of diffuse large B-cell lymphoma.

During a median follow-up of 32.5 months, the high CAR group had significantly poorer 3-year rates of OS (68.3% vs. 96.2%; p < 0.0001) and PFS (53.5% vs. 88.0%; p < 0.0001) (Fig. 1). In the univariable Cox analyses, high CAR values significantly predicted poor OS (HR: 11.22, 95% CI 2.61–48.28; p = 0.001) and poor PFS (HR: 5.68, 95% CI 2.50–12.93; p < 0.001). In addition, most IPI components significantly predicted both OS and PFS, with the exception of > 1 extranodal lesions at the diagnosis (for OS, HR: 2.31, 95% CI 0.98–5.45, p = 0.055; for PFS, HR: 1.85, 95% CI 0.97–3.51, p = 0.061) (Table 2). In the analysis of the GCB subtype, patients with non-GCB revealed poorer tendencies for both OS and PFS, although these were not statistically significant. Patients who received more than 6 cycles of R-CHOP showed significantly worse PFS (HR: 1.97, 95% CI 1.03–3.76, p = 0.040) than patients treated with 6 cycles, However, we did not include the cycles of R-CHOP in multivariate analysis considering possible selection bias. After adjustment for age (> 60 years), stage (≥ III), LDH (> upper normal limit), Eastern Cooperative Oncology Group (ECOG) performance status (> 1), and extranodal involvement (> 1 lesion), high CAR values independently predicted poor OS (HR: 6.02, 95% CI 1.19–30.38; p = 0.030) and poor PFS (HR: 3.62, 95% CI 1.40–9.36; p = 0.008). High CAR was still an independent prognostic marker for PFS after adjusting for the IPI itself, but not for OS (Table 3). When patients were stratified by IPI score (0–2 vs. 3–5), the high CAR group showed significantly worse 3-year PFS (72.7% vs. 91.5%, p = 0.019) in the lower IPI group, although 3-year OS did not show any significant difference (Supplementary Figure S1). In the higher IPI group, the high CAR group presented tendencies toward poorer 3-year OS (53.8% vs. 80.0%, p = 0.059) and PFS (44.0% vs. 70.0%, p = 0.079), although these were not statistically significant (Supplementary Figure S2).

**Table 2:** Univariable analyses of overall survival and progression-free survival in patients with available pre-treatment C-reactive protein-to-albumin ratio data.

	OS			PFS		
	HR	(95% CI)	*p*	HR	(95% CI)	*p*
Age > 60 years	2.81	(1.09–7.27)	***0.033***	2.06	(1.05–4.03)	***0.035***
Stage (III, IV)	5.66	(1.66–19.23)	***0.006***	4.51	(1.98–10.25)	** < 0.001**
LDH > UNL	6.77	(1.57–29.14)	***0.010***	3.19	(1.40–7.25)	***0.006***
ECOG PS > 1	4.14	(1.67–10.29)	***0.002***	3.49	(1.69–7.21)	***0.001***
EN > 1	2.31	(0.98–5.45)	0.055	1.85	(0.97–3.51)	0.061
CAR > 0.158	11.22	(2.61–48.28)	***0.001***	5.68	(2.50–12.93)	** < 0.001**
Cycles of R-CHOP > 6^a^	2.14	(0.72–6.38)	0.173	1.97	(1.03–3.76)	***0.040***
Non-GCB type^b^	3.26	(0.76–13.91)	0.110	1.90	(0.85–4.23)	0.118

*OS* overall survival, *PFS* progression-free survival, *HR* hazard ratio, *CI* confidence interval, *LDH* lactate dehydrogenase, *UNL* upper normal limit, *ECOG PS* Eastern Cooperative Oncology Group performance status, *EN* extranodal, *CAR* C-reactive protein-to-albumin ratio, *GCB* germinal centre B-cell.

^a^Compared to 6.

^b^Compared to GCB type.

**Table 3:** Multivariable analyses of overall survival and progression-free survival in patients with available pre-treatment C-reactive protein-to-albumin ratio data.

	OS			PFS		
	HR	(95% CI)	*p*	HR	(95% CI)	*p*
Age > 60 years	2.91	(1.05–8.07)	0.040	1.90	(0.93–3.89)	***0.080***
Stage (III, IV)	2.15	(0.54–8.57)	0.278	2.52	(1.00–6.36)	***0.050***
LDH > UNL	1.77	(0.34–9.19)	0.499	1.16	(0.44–3.05)	0.769
ECOG PS > 1	1.96	(0.77–4.97)	0.158	1.81	(0.85–3.84)	0.124
EN > 1	1.26	(0.48–3.35)	0.637	0.96	(0.46–1.98)	0.908
CAR > 0.158	6.02	(1.19–30.38)	***0.030***	3.62	(1.40–9.36)	***0.008***
IPI ≥ 3	8.97	(1.93–41.63)	***0.005***	3.17	(1.43–7.00)	***0.004***
CAR > 0.158	4.35	(0.94–20.15)	0.060	3.35	(1.36–8.20)	***0.008***

*OS* overall survival, *PFS* progression-free survival, *HR* hazard ratio, *CI* confidence interval, *LDH* lactate dehydrogenase, *UNL* upper normal limit, *ECOG PS* Eastern Cooperative Oncology Group performance status, *EN* extranodal, *CAR* C-reactive protein-to-albumin ratio, *IPI* international prognostic index.

**Figure 2:**
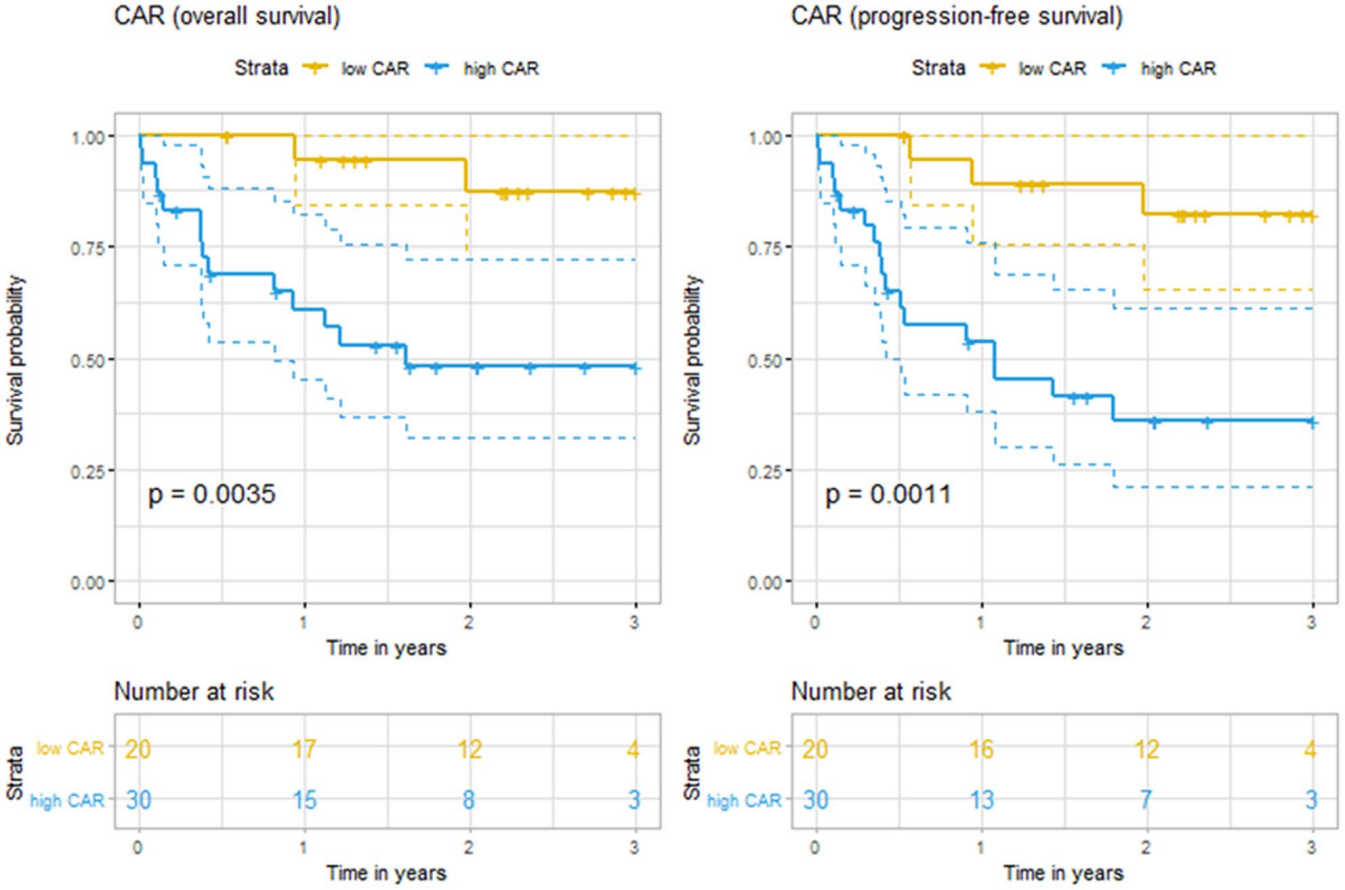
Kaplan–Meier curves for overall survival and progression-free survival according to the C-reactive protein-to-albumin ratio (CAR) at the diagnosis of diffuse large B-cell lymphoma in the validation cohort.

To validate the clinical universality of CAR, we applied the same cut-off value to the independent cohort (n = 50). Males were 52.0% of the group, and 37 (74%) were older than age 60 at diagnosis. CR was achieved in 35 (70%), PR in 6 (12.0%). During a median follow-up of 27 months, the high CAR group showed significantly worse 3-year OS (47.9% vs. 87.2%, p = 0.0035) and 3-year PFS (36.1% vs. 82.1%, p = 0.0011) (Fig. 2). In the univariable Cox analysis, high CAR values were significantly associated with poorer OS (HR: 6.81, 95% CI 1.54–30.11; p = 0.011) and PFS (HR: 6.07, 95% CI 1.77–20.85; p = 0.004). After adjustment for the components of the IPI, the high CAR values retained their predictability for OS (HR: 6.13, 95% CI 1.06–35.34; p = 0.042) and PFS (HR: 4.66, 95% CI 1.09–19.93; p = 0.038) (Supplementary Table S1).

### Prognostic value of the CAR according to age

**Figure 3:**
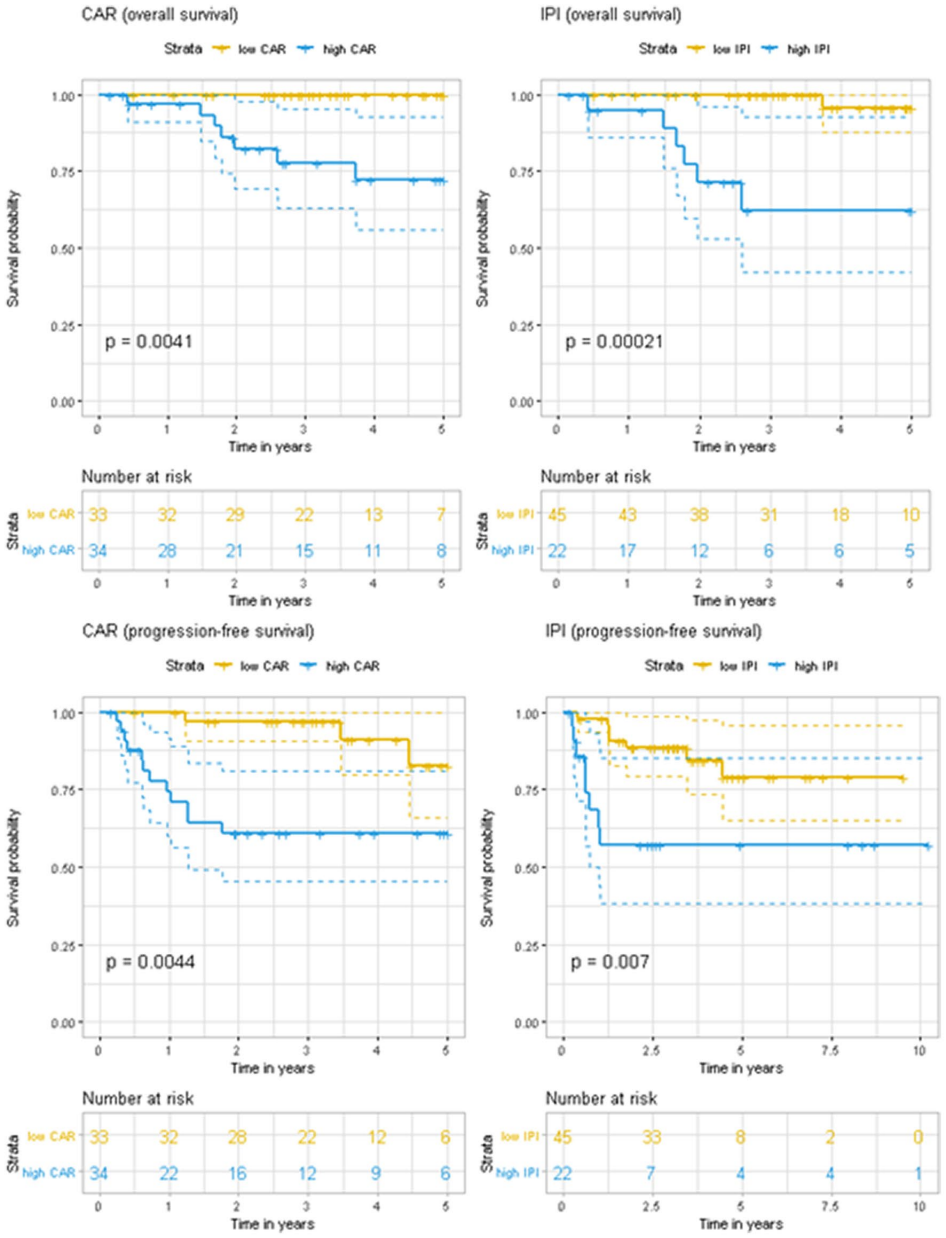
Kaplan–Meier curves for overall survival and progression-free survival according to the C-reactive protein-to-albumin ratio (CAR) and International Prognostic Index (IPI) among patients who were ≤ 60 years old at the diagnosis of diffuse large B-cell lymphoma.

**Figure 4:**
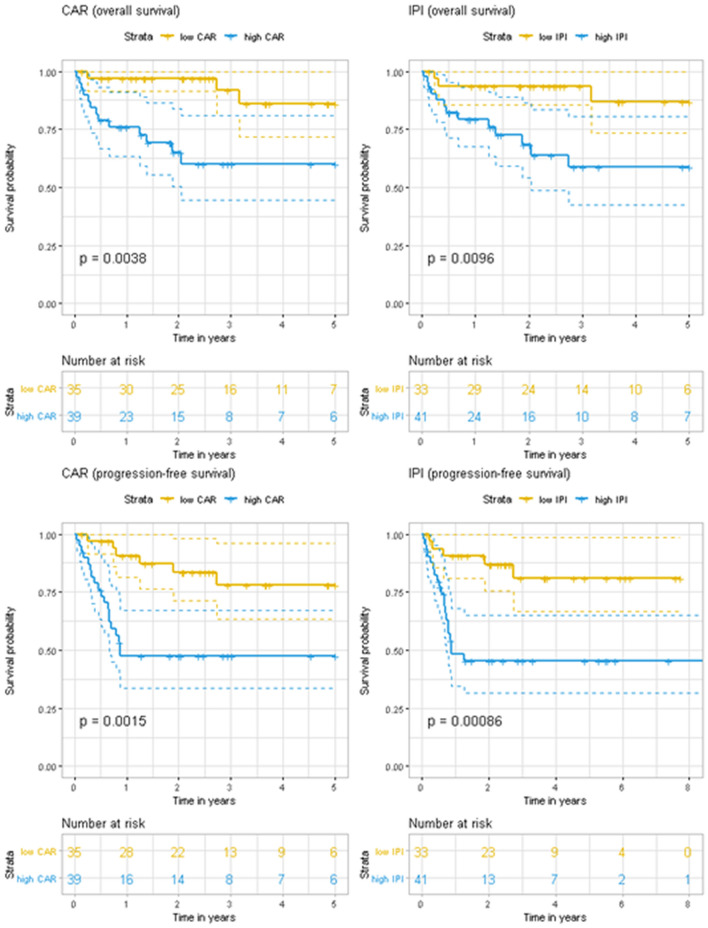
Kaplan–Meier curves for overall survival and progression-free survival according to the C-reactive protein-to-albumin ratio (CAR) and International Prognostic Index (IPI) among patients who were > 60 years old at the diagnosis of diffuse large B-cell lymphoma.

**Figure 5:**
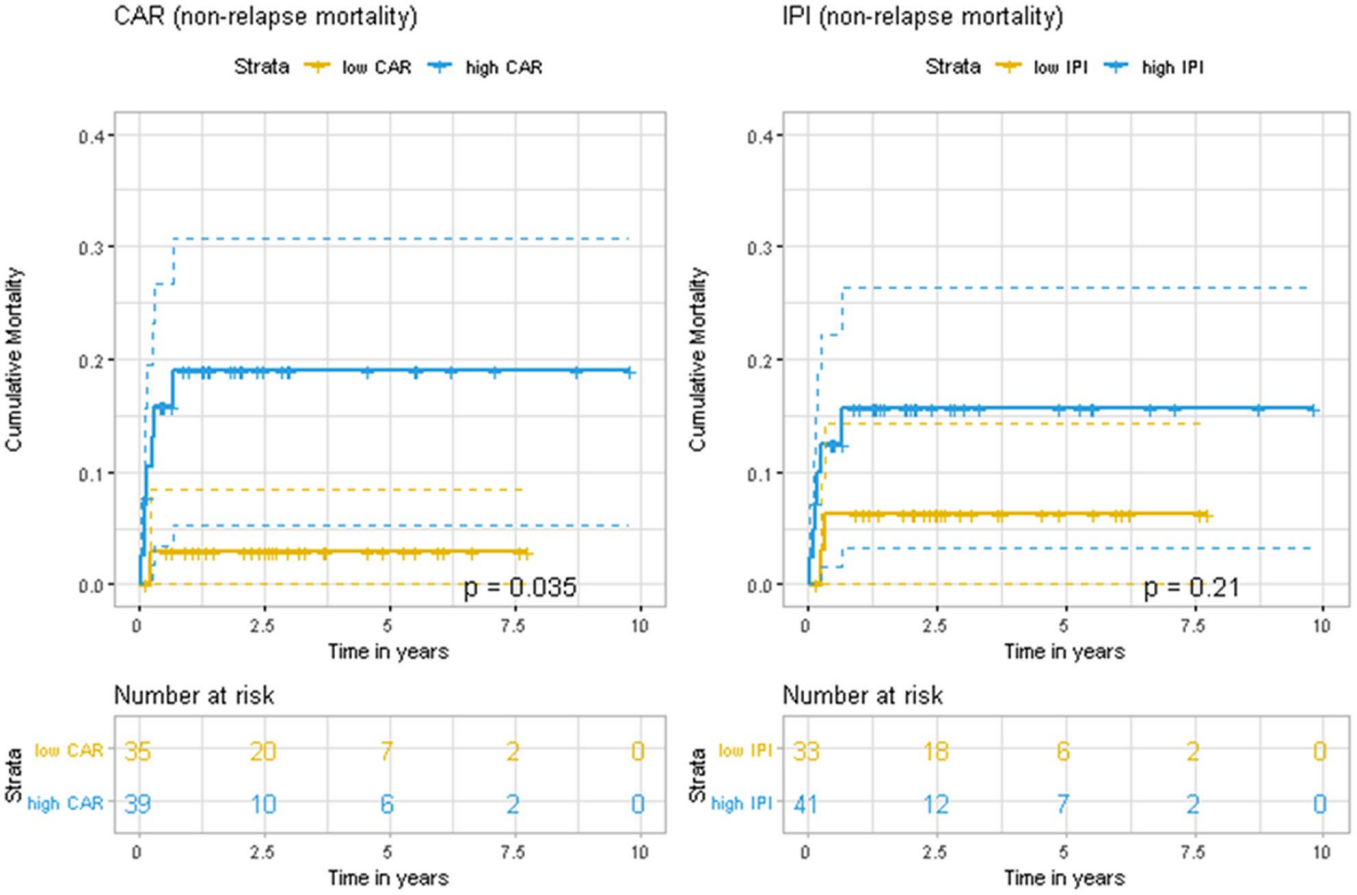
Kaplan–Meier curves for non-relapse mortality according to the C-reactive protein-to-albumin ratio (CAR) and International Prognostic Index (IPI) among patients who were > 60 years old at the diagnosis of diffuse large B-cell lymphoma.

Subgroup analyses were performed to determine whether the prognostic value of CAR varied according to age. Among the 65 patients who were ≤ 60 years old and had available CAR data, we observed that high CAR and high IPI (≥ 3) values significantly predicted poor 3-year OS (for CAR: 77.6% vs. 100%, p = 0.0041; for IPI: 62.3% vs. 100.0%, p = 0.00021) and poor 3-year PFS (for CAR: 60.7% vs. 96.8%, p = 0.0044; for IPI: 57.0% vs. 88.3%, p = 0.007) (Fig. 3). Among the 74 patients who were > 60 years old at the diagnosis, high CAR and high IPI values also significantly predicted poor 3-year OS (for CAR: 60.0% vs. 91.7%, p = 0.0038; for IPI: 58.6% vs. 93.8%, p = 0.0096) and poor 3-year PFS (for CAR: 47.6% vs. 77.9%, p = 0.0015; for IPI: 45.3% vs. 81.1%, p = 0.00086) (Fig. 4). Among the older patients, a high CAR value also significantly predicted a poor NRM rate (p = 0.035), although a high IPI value did not significantly predict a poor NRM rate (p = 0.21) (Fig. 5). None of the younger patients (< 60 years old at diagnosis) experienced NRM. In validation cohort, a high CAR value did not predict a poor NRM rate in both all patients (p = 0.12) and elderly patients (p = 0.26) despite of tendencies toward poor outcome (Supplementary Figure S3).

### Exploring other non-CAR laboratory biomarkers

The prognostic value of the AGR was compared between the high and low AGR groups (> 1.381 vs. ≤ 1.381). Relative to the low AGR group (n = 72), the high AGR group (n = 114) experienced significantly better 3-year OS (89.1% vs. 75.6%, p = 0.017) and tended to have more favourable 3-year PFS (73.2% vs. 63.2%, p = 0.068) (Supplementary Figure S4). In the univariable Cox analyses, high AGR was significantly associated with superior OS (HR: 0.40, 95% CI 0.18–0.87; p = 0.022) but not with superior PFS (HR: 0.61, 95% CI 0.35–1.04, p = 0.071). After adjustment for the IPI components, AGR was not significantly related to OS (HR: 0.68, 95% CI: 0.30–1.51; p = 0.338) or PFS (HR: 0.95, 95% CI 0.54–1.68; p = 0.869) (Supplementary Table S2).

Serum ferritin data were available for 83 patients (> 319 ng/mL vs. ≤ 319 ng/mL). Relative to the low ferritin group, the high ferritin group (n = 17) experienced significantly poorer 3-year OS (55.5% vs. 86.0%, p = 0.027) but not significantly poorer 3-year PFS (61.1% vs. 68.6%, p = 0.45) (Supplementary Figure S5). In the univariable Cox analysis, high serum ferritin was significantly associated with poor OS (HR: 3.41, 95% CI 1.07–10.89; p = 0.038), although serum ferritin was not independently associated with OS or PFS in the multivariable Cox analyses (Supplementary Table S3).

Serum beta 2 microglobulin data were available for 121 patients (> 3.09 mg/L vs. ≤ 3.09 mg/L). Relative to the low beta 2 microglobulin group, the high beta 2 microglobulin group (n = 28) experienced significantly worse 3-year OS (70.5% vs. 89.3%, p = 0.011) and 3-year PFS (48.9% vs. 74.8%, p = 0.0068) (Supplementary Figure S6). In the univariable Cox analyses, high serum beta 2 microglobulin was significantly associated with poor OS (HR: 3.66, 95% CI 1.25–10.71; p = 0.018) and poor PFS (HR: 2.48, 95% CI 1.26–4.88; p = 0.009). In the multivariable Cox analyses, high beta 2 microglobulin levels did not predicted poor OS or PFS (Supplementary Table S4).

## Discussion

This retrospective study evaluated the prognostic value of CAR and other markers in patients with DLBCL and revealed three main findings. First, a high pre-treatment CAR significantly predicted poor OS and poor PFS. Second, it appears that high CAR values, especially in older patients, may predict NRM, in addition to OS and PFS. Third, AGR, serum ferritin, and beta 2 microglobulin were potentially useful prognostic markers, although they did not remain significant after adjustment for the IPI components. To the best of our knowledge, this is the first study to examine the prognostic value of CAR in haematological malignancies.

Recent research has focused on the use of immune markers and their ability to predict clinical outcomes. For example, CRP is one of the strongest markers of the innate immune system, and is considered a clinically and pathologically significant acute-phase marker of infection and inflammation^24^. In addition, CRP has prognostic value in various malignancies and can be used to predict treatment response and tumour recurrence^15,25^. Serum albumin has also been recognised as a clinically significant nutritional marker, and may be a useful tumour marker, as tumour cells might produce higher rates of albumin degradation and turnover^26^. Another report has indicated that low serum albumin levels were associated with the inflammatory process, which might influence the response to anticancer therapy via effects on drug pharmacokinetics and adverse drug reactions^27^. These markers (CRP and albumin) have been examined for creating risk groups of patients with DLBCL^28^, and the prognostic value of the CAR has also be considered based on the changing CRP and albumin levels in the tumour microenvironment^21^. The present study revealed that the pre-treatment CAR value could be used to predict clinical outcomes and that this prognostic value was independent of the IPI components. Moreover, the pre-treatment CAR value was able to robustly predict diverse clinical outcomes (including NRM) in elderly patients, who are a unique subgroup with many comorbidities. Thus, given that CAR considers relatively non-specific biomarkers, relative to the cancer-specific IPI components, it is possible that the CAR could be useful for predicting more diverse clinical outcomes. In addition, a high CAR might predict NRM in elderly patients during induction therapy, so we recommend closer monitoring and active supportive care for this group.

The present study also evaluated the prognostic values of pre-treatment AGR, serum ferritin, and beta 2 microglobulin. In this context, pre-treatment AGR has been suggested as a prognostic marker in various diseases, including DLBCL^29,30^, while serum ferritin and beta 2 microglobulin have also been proposed to have prognostic value in patients with DLBCL^31–34^. The present study revealed some prognostic value for these markers in the univariate analyses, although most of the relationships disappeared after adjusting for the IPI components, with the exception of the relationship between beta 2 microglobulin and OS. Nevertheless, higher values for these markers tended to be associated with a relatively poor prognosis, and our findings are generally compatible with those of previous studies that described these markers’ roles in inflammation and/or tumour burden^35–37^.

The present study has several limitations that should be addressed. First, although we included a meaningful number of patients (n = 141), a relatively high percentage of cases (24%, 45/186) were excluded because CRP was not a routine work-up at diagnosis. In addition, while 25 deaths occurred during the follow-up period, 25 patients were also lost to follow-up. These potential limitations (high rates of exclusion and loss to follow-up) are attributable to the retrospective design, and further validation in a prospective multicentre study is needed. However, we included all patients who were available in our institution. In addition, we also tested the clinical validity of CAR in an independent cohort. In this aspect, our results have value despite the relatively small sample size. Second, we only included patients who received standard R-CHOP therapy in an attempt to avoid confounding, although this precluded an analysis of salvage therapy after relapse and/or HSCT, which are important factors that affect survival outcomes^4,38^. However, our centre tends to use a standardised salvage regimen and policy for HSCT in patients with DLBCL, which would suggest that these factors did not substantially affect our findings. Nevertheless, it would be prudent to consider changes in these biomarkers based on any changes to the therapeutic regimen and in patients who received HSCT. Third, the IPI remains the strongest prognostic index for patients with DLBCL, and none of the potential inflammatory markers have been able to exceed the robustness of the IPI. While the present study aimed to identify novel and robust prognostic markers, the IPI remained a powerful marker, as expected. Although, CAR did not outperform IPI in terms of prognostication, our findings revealed that CAR might be another useful prognostic marker in DLBCL, especially for predicting NRM in elderly patients although it was not significant when we independently validated probably due to small sample size. Thus, given the convenience and objectivity of the CAR, further studies are needed to validate its prognostic significance in this setting.

In conclusion, the pre-treatment CAR might be a prognostic marker for DLBCL, especially in cases involving elderly patients. Furthermore, the AGR, serum ferritin, and beta 2 microglobulin markers exhibited some potential prognostic value. Therefore, further studies are needed to verify the precise prognostic values of these markers in this setting.

## Supplementary Information

Supplementary Information

## Data Availability

All relevant data are included in the manuscript and the supplementary information.
