# Supplementary material for: High level of pre-treatment C-reactive protein to albumin ratio predicts inferior prognosis in diffuse large B-cell lymphoma
Source: Sci Rep. 2021 Jan 29;11:2674. doi: 10.1038/s41598-021-82087-6 (PMC7846592; doi:10.1038/s41598-021-82087-6)
Supplement: Supplementary file 1 — Supplementary Information [file 41598_2021_82087_MOESM1_ESM.pdf]

High Level of Pre-treatment C-reactive Protein to Albumin Ratio Predicts Inferior Prognosis in Diffuse Large B-cell Lymphoma

Jongheon Jung, Hyewon Lee, Ja Yoon Heo, Myung Hee Chang, Eunyoung Lee, Weon Seo Park, Ju-Hyun Park, Hyeon-Seok Eom

SUPPLEMENTARY INFORMATION

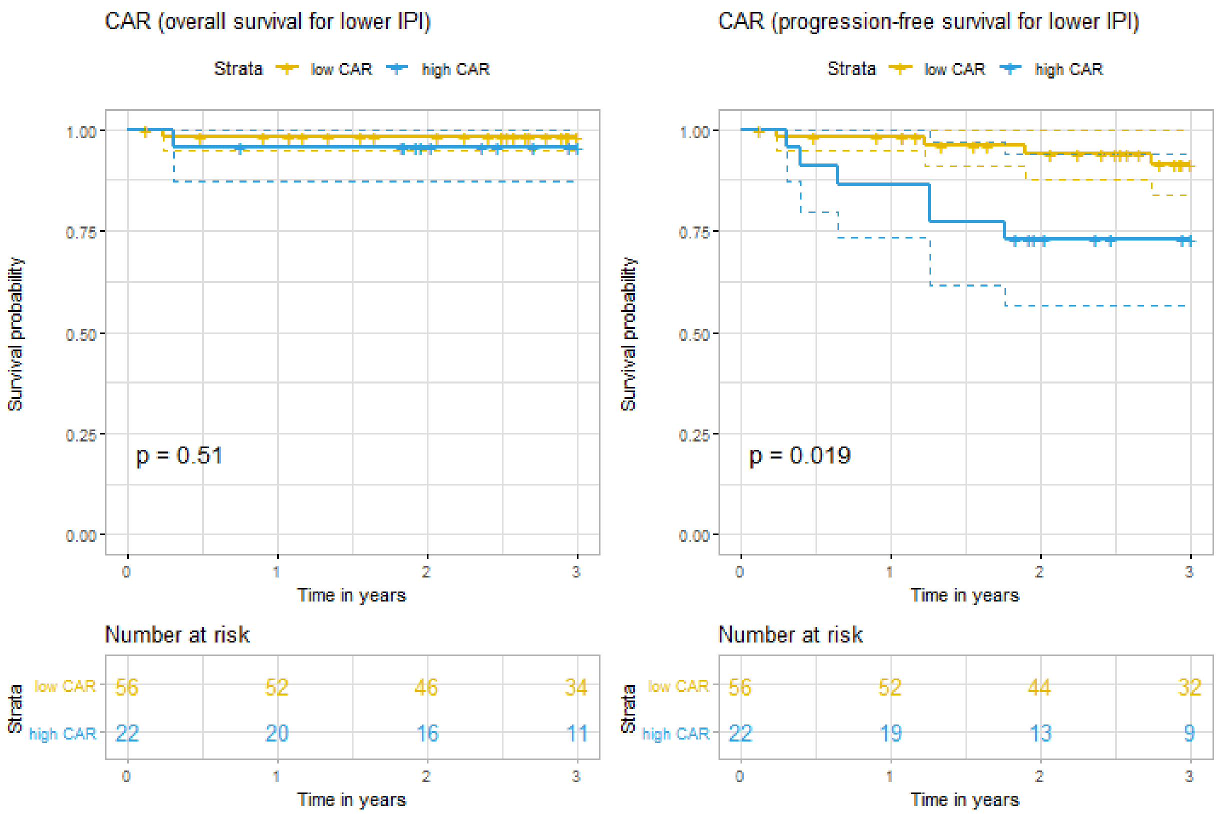

Figure S-1. Kaplan–Meier curves for overall survival and progression-free survival according to the C-reactive protein-to-albumin ratio (CAR) at the diagnosis of diffuse large B-cell lymphoma in patients with lower International Prognostic Index (0–2).

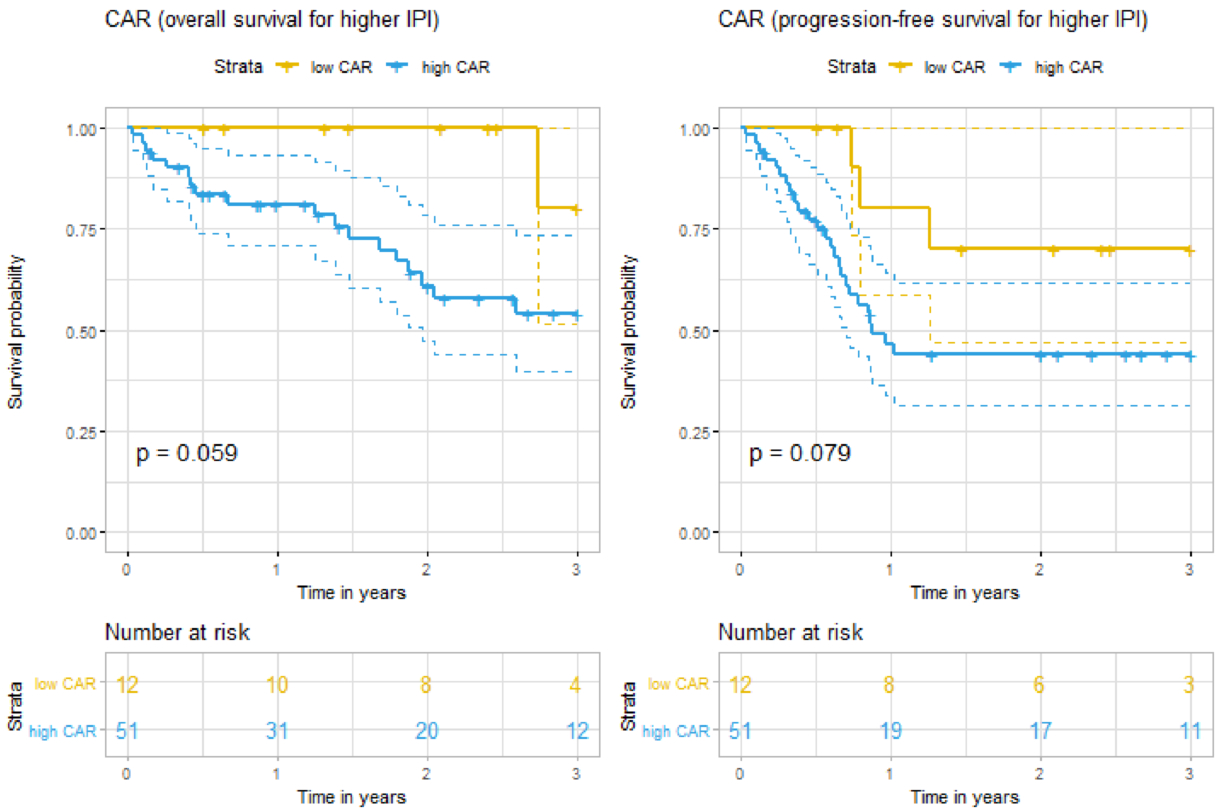

Figure S-2. Kaplan–Meier curves for overall survival and progression-free survival according to the C-reactive protein-to-albumin ratio (CAR) at the diagnosis of diffuse large B-cell lymphoma in patients with higher International Prognostic Index (3–5).

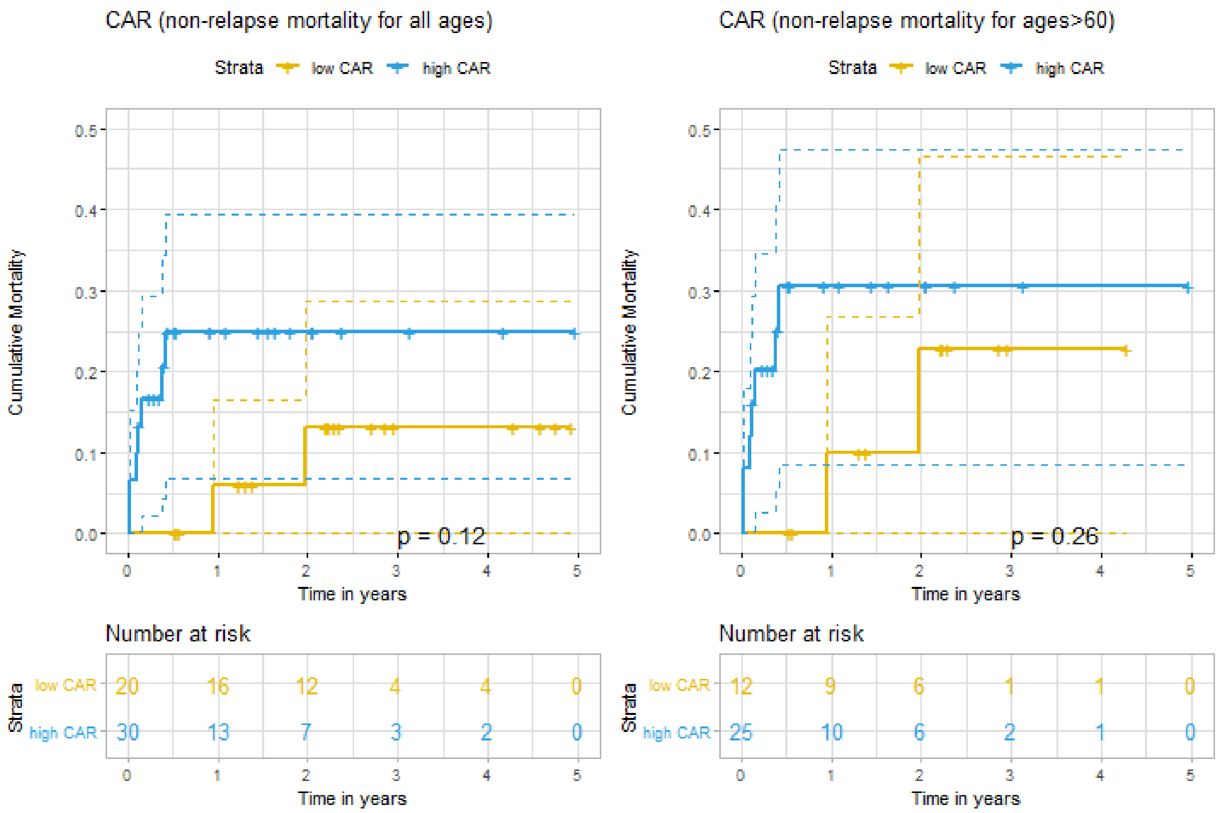

Figure S-3. Kaplan–Meier curves for non-relapse mortality according to the C-reactive protein-to-albumin ratio (CAR) at the diagnosis of diffuse large B-cell lymphoma in the validation cohort.

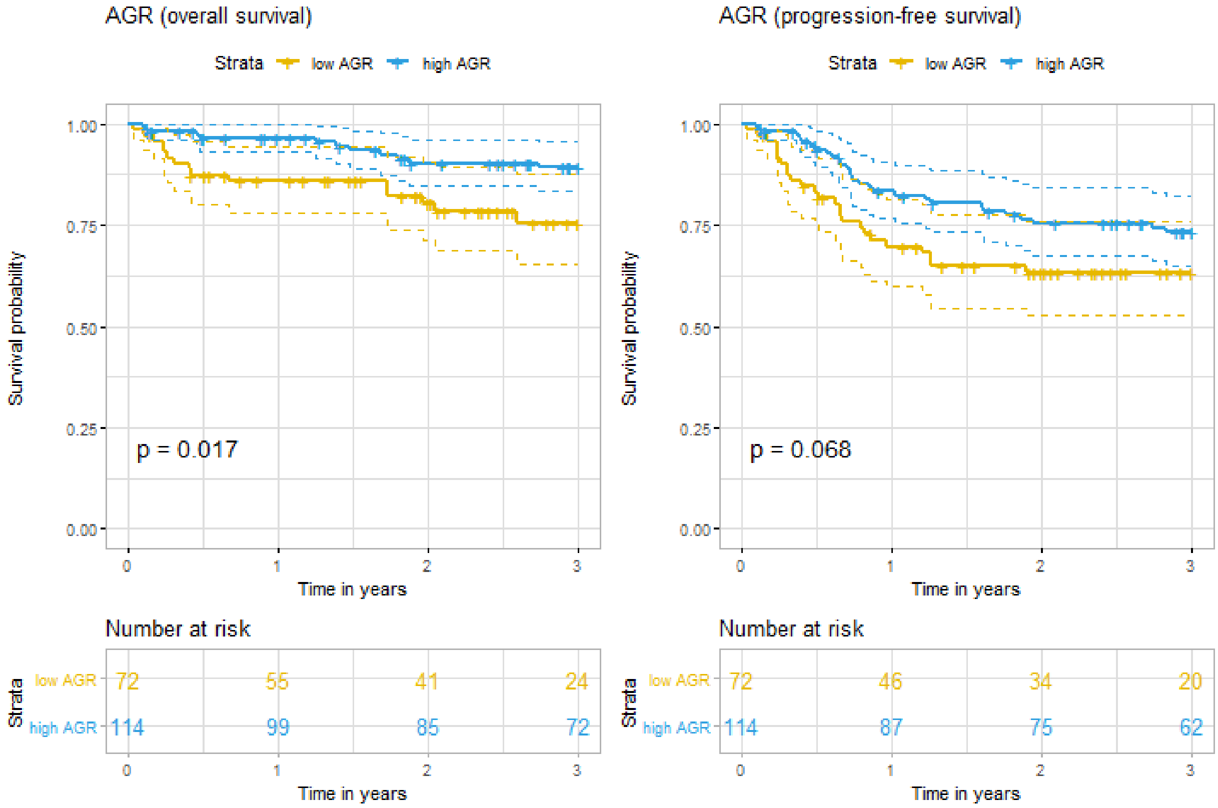

Figure S-4. Kaplan–Meier curves for overall survival and progression-free survival according to the albumin-to-globulin ratio (AGR) at the diagnosis of diffuse large B-cell lymphoma.

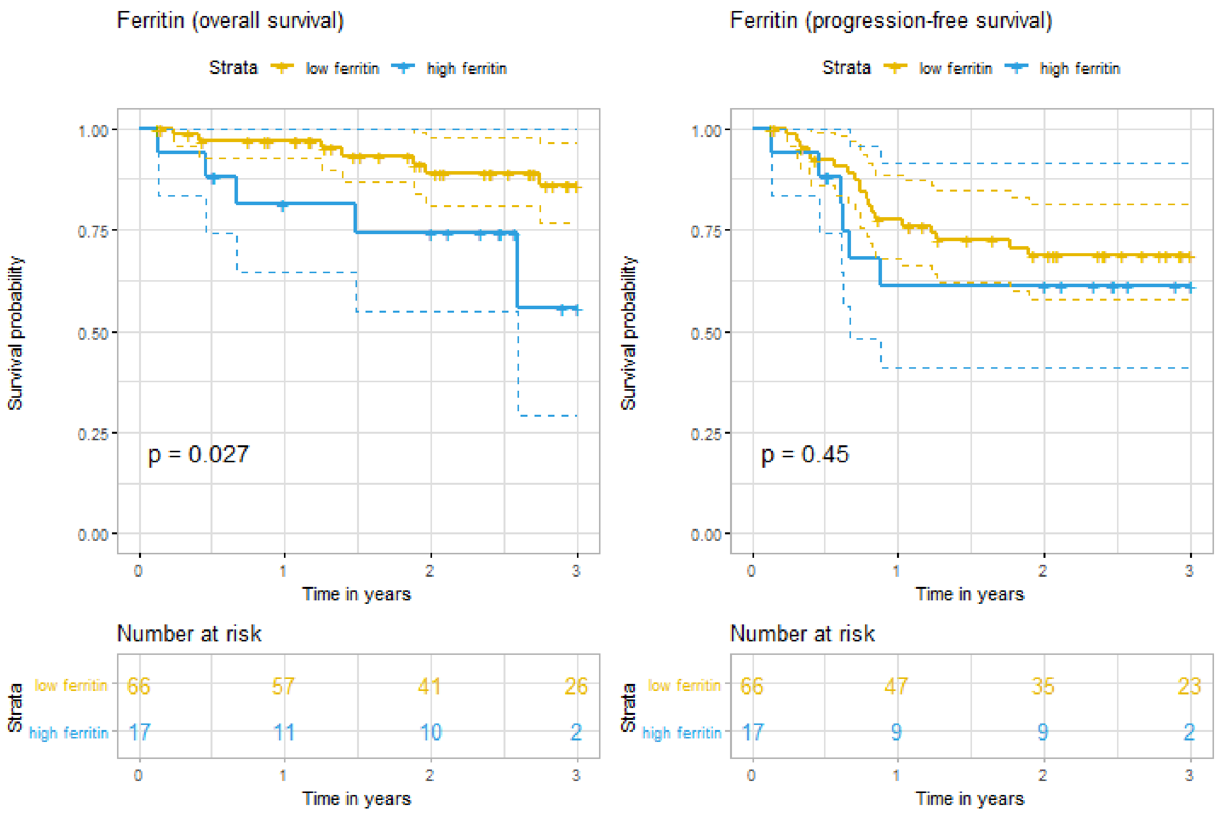

Figure S-5. Kaplan–Meier curves for overall survival and progression-free survival according to serum ferritin levels at the diagnosis of diffuse large B-cell lymphoma.

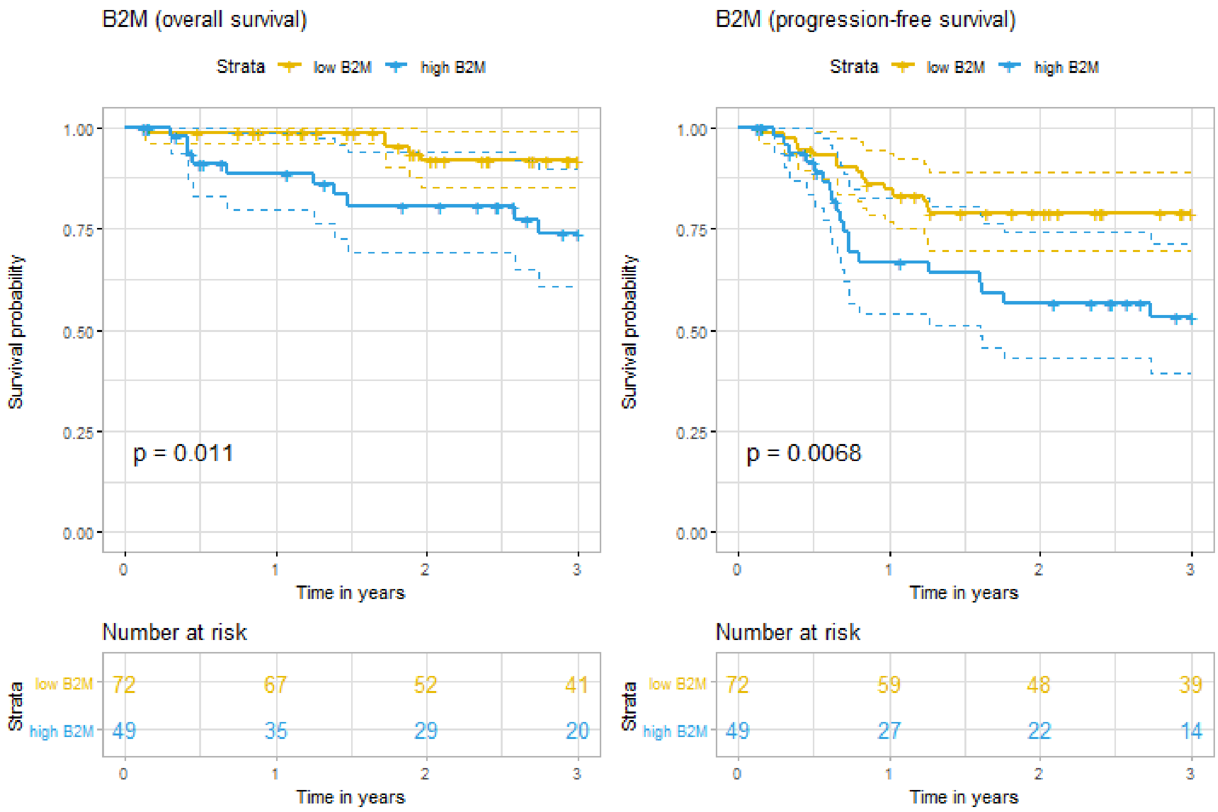

Figure S-6. Kaplan–Meier curves for overall survival and progression-free survival according to serum beta 2 microglobulin (B2M) levels at the diagnosis of diffuse large B-cell lymphoma.

Table S-1. Clinical characteristics of patients in validation cohort

	Total	Low CAR (≤0.158)	High CAR (>0.158)	P*	P†
**Number**	50	20 (40.0%)	30 (60.0%)		
**Male sex**	26 (52.0%)	9 (45.0%)	17 (56.7%)	0.419	0.611
**Age >60 years**	37 (74.0%)	12 (60.0%)	25 (83.3%)	0.065	0.129
**Stage >2**	28 (56.0%)	7 (35.0%)	21 (75.0%)	***0.015***	0.750
**ECOG PS >1**	22 (44.0%)	3 (15.0%)	19 (63.3%)	***0.001***	0.627
**LDH >UNL**	22 (44.0%)	5 (25.0%)	17 (56.7%)	***0.027***	0.079
**Extranodal >1**	18 (36.0%)	5 (25.0%)	13 (43.3%)	0.186	0.430
**IPI**				***0.037***	0.183
**0**	5 (10.0%)	4 (20.0%)	1 (3.3%)		
**1**	10 (20.0%)	6 (30.0%)	4 (13.3%)		
**2**	9 (18.0%)	5 (25.0%)	4 (13.3%)		
**3**	9 (18.0%)	2 (10.0%)	7 (23.3%)		
**4**	11 (22.0%)	3 (15.0%)	8 (26.7%)		
**5**	6 (12.0%)	0 (0%)	6 (20.0%)		
**Non-GCB type**	47 (94.0%)	19 (95.0%)	28 (93.3%)	1.000	0.428
**Cycles of R-CHOP**	6 [1-8]	6 [4-6]	6 [1-8]	0.090	0.288
**Response to induction**					
**CR**	35 (70.0%)	17 (85.0%)	18 (60.0%)	0.180	***0.031***
**PR**	6 (12.0%)	2 (10.0%)	4 (13.3%)		
**SD**	0 (0%)	0 (0%)	0 (0%)		
**PD**	4 (8.0%)	1 (5.0%)	3 (10.0%)		
**Not available**	5 (10.0%)	0 (0%)	5 (16.7%)		
**Overall response**	41 (91.1%)	19 (95.0%)	22 (88.0%)	0.617	0.082
**CRP (mg/dL)**	1.46 [0.20-9.91]	0.50 [0.20-0.66]	3.69 [0.50-9.91]	***<0.001***	0.999
**Albumin (g/dL)**	3.7 [1.9-4.7]	4.2 [3.5-4.7]	3.5 [1.9-4.1]	***<0.001***	0.999

Data are presented as number of patients (frequency) for categorical variables and as median [range] for continuous variables.

*High-CAR group vs. low-CAR group. †compared between the original cohort and validation cohort

CAR, C-reactive protein-to-albumin ratio; ECOG PS, Eastern Cooperative Oncology Group performance status; LDH, lactate dehydrogenase; UNL, upper normal limit; IPI, International Prognostic Index; GCB, germinal centre B-cell; R-CHOP, rituximab, cyclophosphamide, doxorubicin, vincristine, and prednisone; CR, complete response; PR, partial response; SD, stable disease; PD, progressive disease

Table S-2. Univariable and multivariable analyses of overall survival and progression-free survival in validation cohort

	Univariable OS			Multivariable OS		
	HR	(95% CI)	*p*	HR	(95% CI)	*p*
Age >60 years*	NA	NA	NA	NA	NA	NA
Stage (III, IV)	2.95	(0.95–9.18)	0.062	0.86	(0.18–4.21)	0.855
LDH >UNL	2.04	(0.58–7.17)	0.266	1.58	(0.26–9.53)	0.617
ECOG PS >1	2.89	(1.04–8.00)	***0.041***	0.70	(0.16–3.01)	0.628
EN >1	3.28	(1.19–9.03)	***0.022***	2.16	(0.47–9.97)	0.323
CAR >0.158	6.81	(1.54–30.11)	***0.011***	6.13	(1.06–35.34)	***0.042***
	**Univariable PFS**			**Multivariable PFS**		
	HR	(95% CI)	*p*	HR	(95% CI)	*p*
Age >60 years	4.28	(0.99–18.49)	0.051	2.66	(0.57–12.53)	0.215
Stage (III, IV)	3.22	(1.16–8.90)	***0.024***	1.08	(0.27–4.26)	0.911
LDH >UNL	2.78	(0.81–9.51)	0.103	1.61	(0.32–8.13)	0.562
ECOG PS >1	3.60	(1.42–9.11)	***0.007***	0.97	(0.26–3.56)	0.959
EN >1	3.29	(1.34–8.06)	***0.009***	1.96	(0.54–7.12)	0.306
CAR >0.158	6.07	(1.77–20.85)	***0.004***	4.66	(1.09–19.93)	***0.038***

OS, overall survival; PFS, progression-free survival; HR, hazard ratio; CI, confidence interval; UNL, upper normal limit; ECOG PS, Eastern Cooperative Oncology Group performance status; EN, extranodal; CAR, C-reactive protein-to-albumin ratio

*no death in patients ≤60 years

Table S-3. Univariable and multivariable analyses of overall survival and progression-free survival in patients with available pre-treatment albumin-to-globulin ratio data

	**Univariable OS**			**Multivariable OS**		
	HR	(95% CI)	*p*	HR	(95% CI)	*p*
Age >60 years	1.90	(0.85–4.28)	0.118	1.60	(0.70–3.65)	0.261
Stage (III, IV)	3.86	(1.46–10.25)	***0.007***	1.99	(0.66–5.95)	0.221
LDH >UNL	8.90	(2.10–37.69)	***0.003***	5.65	(1.27–25.22)	***0.023***
ECOG PS >1	3.72	(1.56787–8.87)	***0.003***	2.09	(0.85–5.18)	0.109
EN >1	2.43	(1.12–5.25)	***0.024***	1.09	(0.46–2.60)	0.848
AGR >1.381	0.40	(0.18–0.87)	***0.022***	0.68	(0.30–1.51)	0.338
	**Univariable PFS**			**Multivariable PFS**		
	HR	(95% CI)	*p*	HR	(95% CI)	*p*
Age >60 years	2.15	(1.20–3.82)	***0.010***	1.83	(1.01–3.31)	***0.046***
Stage (III, IV)	5.03	(2.45–10.32)	***<0.001***	3.57	(1.62–7.87)	***0.002***
LDH >UNL	2.91	(1.50–5.66)	***0.002***	1.76	(0.86–3.58)	0.121
ECOG PS >1	3.04	(1.56–5.91)	***0.001***	1.72	(0.86–3.43)	0.124
EN >1	2.39	(1.39–4.09)	***0.002***	1.12	(0.62–2.02)	0.715
AGR >1.381	0.61	(0.35–1.04)	0.071	0.95	(0.54–1.68)	0.869

OS, overall survival; PFS, progression-free survival; HR, hazard ratio; CI, confidence interval; UNL, upper normal limit; ECOG PS, Eastern Cooperative Oncology Group performance status; EN, extranodal; AGR, albumin-to-globulin ratio

Table S-4. Univariable and multivariable analyses of overall survival and progression-free survival in patients with available pre-treatment serum ferritin levels

	Univariable OS			Multivariable OS		
	HR	(95% CI)	*p*	HR	(95% CI)	*p*
Age >60 years	4.50	(1.21–16.79)	***0.025***	4.64	(1.12–19.18)	***0.034***
Stage (III, IV)	4.51	(0.98–20.67)	0.053	3.37	(0.66–17.16)	0.143
LDH >UNL	5.69	(0.73–44.15)	0.096	2.94	(0.34–25.05)	0.325
ECOG PS >1	2.13	(0.58–7.86)	0.258	0.69	(0.15–3.24)	0.640
EN >1	1.26	(0.40–3.99)	0.693	0.67	(0.19–2.27)	0.516
Ferritin >319 ng/mL	3.41	(1.07–10.89)	***0.038***	2.58	(0.67–10.00)	0.169

	Univariable PFS			Multivariable PFS		
	HR	(95% CI)	*p*	HR	(95% CI)	*p*
Age >60 years	2.89	(1.24–6.73)	***0.014***	2.62	(1.08–6.32)	0.032
Stage (III, IV)	3.65	(1.37–9.75)	***0.010***	3.34	(1.18–9.48)	***0.024***
LDH >UNL	2.78	(0.95–8.10)	***0.062***	2.07	(0.67–6.41)	0.207
ECOG PS >1	1.38	(0.47–4.02)	0.556	0.68	(0.21–2.24)	0.528
EN >1	1.44	(0.65–3.17)	0.369	1.00	(0.41–2.41)	0.997
Ferritin >319 ng/mL	1.42	(0.57–3.57)	0.451	0.91	(0.33–2.53)	0.853

OS, overall survival; PFS, progression-free survival; HR, hazard ratio; CI, confidence interval; LDH, lactate dehydrogenase; UNL, upper normal limit; ECOG PS, Eastern Cooperative Oncology Group performance status; EN, extranodal.

Table S-5. Univariable and multivariable analyses of overall survival and progression-free survival in patients with available pre-treatment serum beta 2 microglobulin levels

	Univariable OS			Multivariable OS		
	HR	(95% CI)	*p*	HR	(95% CI)	*p*
Age >60 years	1.90	(0.67–5.34)	0.225	1.74	(0.58–5.26)	0.323
Stage (III, IV)	2.80	(0.89–8.82)	0.078	1.42	(0.39–5.21)	0.597
LDH >UNL*	NA	NA	NA	NA	NA	NA
ECOG PS >1	2.20	(0.62–7.82)	0.221	1.00	(0.26–3.92)	0.996
EN >1	1.56	(0.55–4.39)	0.399	0.67	(0.21–2.10)	0.487
B2M >3.09 mg/L	3.66	(1.25–10.71)	***0.018***	2.10	(0.66–6.72)	0.211

	Univariable PFS			Multivariable PFS		
	HR	(95% CI)	*p*	HR	(95% CI)	*p*
Age >60 years	1.98	(0.99–3.97)	0.053	1.73	(0.83–3.61)	0.144
Stage (III, IV)	3.57	(1.61–7.89)	***0.002***	2.45	(1.01–5.95)	***0.048***
LDH >UNL	3.61	(1.49–8.74)	***0.004***	2.56	(1.02–6.46)	***0.046***
ECOG PS >1	2.56	(1.12–5.90)	***0.027***	1.31	(0.53–3.25)	0.555
EN >1	1.77	(0.89–3.50)	0.103	0.89	(0.42–1.89)	0.770
B2M >3.09 mg/L	2.48	(1.26–4.88)	***0.009***	1.34	(0.63–2.86)	0.443

OS, overall survival; PFS, progression-free survival; HR, hazard ratio; CI, confidence interval; LDH, lactate dehydrogenase; UNL, upper normal limit; ECOG PS, Eastern Cooperative Oncology Group performance status; EN, extranodal; B2M, beta 2 microglobulin.

*no death in patients with normal LDH.
